# Synergetic protective effect of berberine and ginsenoside Rb1 against tumor necrosis factor alpha−induced inflammation in adipocytes

**DOI:** 10.1080/21655979.2021.1996508

**Published:** 2021-12-04

**Authors:** Zhixing Cai, Yue Chen

**Affiliations:** Department of Traditional Chinese Medicine, Tongren Hospital, Shanghai, China

**Keywords:** Berberine, ginsenoside Rb1, obesity, nuclear factor kappa B, inflammation, adipocyte

## Abstract

Obesity significantly impacts living a normal life by increasing morbidity. Additionally, obesity has been shown to be closely associated with severe inflammation in adipocytes. It is widely reported that berberine (BBR) has an anti-inflammatory effect and can reduce glucose and lipid accumulation, whereas ginsenoside Rb1 (Rb1) has been shown to have a significant inhibitory effect on insulin resistance and lipid peroxidation. In this study, we aimed to explore the synergetic effect of BBR and Rb1 on tumor necrosis factor alpha (TNF-α)-treated adipocytes and the mechanisms underlying it. We found that TNF-α reduced cell viability, facilitated the production of inflammatory factors, induced adipogenesis, activated the nuclear factor kappa B (NF-κB) pathway, and increased the expression of peroxisome proliferator-activated receptor gamma, CCAAT enhancer-binding protein alpha, and sterol regulatory element-binding protein-1 c in adipocytes. However, these effects were significantly alleviated by BBR or Rb1. Additionally, a synergetic effect was observed when BBR and Rb1 were used in combination. The effects of BBR in combination with Rb1 on cell proliferation, inflammation, adipogenesis, and the NF-κB pathway in TNF-α-treated adipocytes were significantly abolished by receptor activator of nuclear factor kappa-Β ligand, which is an activator of the NF-κB pathway. Collectively, the results revealed that BBR and Rb1 have a synergetic protective effect against TNF-α-induced inflammation in adipocytes. The mechanism underlying this synergetic effect was found to be inhibition of the NF-κB signaling pathway.

## Introduction

Obesity is a disease that requires more research attention, as it is associated with significant morbidity. Early in 2003, the number of people who were overweight and obese worldwide were 0.2–0.3 billion and 30–40 million, respectively [1]. The definition of metabolic syndrome was first proposed in 1988 to explain the pathogenesis of related metabolic diseases such as obesity [2]. Metabolic syndrome has been shown to be a chronic inflammatory syndrome, whereas obesity is defined as a mild inflammatory state characterized by upregulation of inflammatory biomarkers. In 1993, an animal experiment showed that tumor necrosis factor alpha (TNF-α) is an important pro-inflammatory factor that is expressed in adipocytes. The expression level of TNF-α was found to be significantly increased in adipocytes isolated from obese animals. Additionally, insulin resistance was considerably blocked by soluble TNF-α receptor immunoglobulin. Furthermore, the expression of plasminogen activation inhibitor-1 is significantly upregulated in adipocytes extracted from obese patients, and this is accompanied by increases in leukocyte count and interleukin (IL)-6 concentration in peripheral blood. It has been shown that TNF-α expression is positively correlated with the body mass index (BMI) of obese patients [3]. An investigation of the relationship between lipid level and inflammation using the Mendelian randomization method revealed that obesity is closely related to fat uptake and the expression of susceptible genes [4]. In addition, it has been indicated that the levels of inflammatory factors such as C-reactive protein are high in obese patients. Therefore, it is important to suppress inflammation in the treatment of obesity and metabolic syndrome.

Berberine (BBR) is an isoquinoline alkaloid that is extracted mainly from *Coptis chinensis* and *Phellodendron chinense*. It is reported that BBR has an anti-inflammatory effect [5] and can suppress glucose and lipid accumulation [6]. The protective effect of BBR against inflammation in adipocytes has been reported previously [7]. BBR has been shown to be a promising anti-obesity agent in multiple clinical studies [8]. Ginsenoside Rb1 (Rb1) is a monomer of ginseng diol saponin that has a significant protective effect on the cardiovascular system and the function of vascular endothelial cells [9]. It is reported that Rb1 can improve insulin resistance, induce hemangiectasis, and suppress lipid peroxidation [10,11]. In addition, it was found in previous studies that the development of steatohepatitis in obese mice is repressed by Rb1 through the regulation of glucolipid metabolism [12,13].

In the present study, we investigated the effects of BBR and Rb1 on TNF-α-induced inflammation in adipocytes to explore the potential synergetic effect of the two drugs on obesity and metabolic syndrome.

## Materials and methods

### Agents, cell culture, and induction of differentiation

BBR and Rb1 were obtained from Sigma-Aldrich (St. Louis, MO, USA). Receptor activator of nuclear factor kappa-Β ligand (RANKL) was purchased from MedChemExpress (Monmouth Junction, NJ, USA). 3T3-L1 preadipocytes were purchased from the American Type Culture Collection (Manassas, VA, USA) and cultured in high-glucose Dulbecco’s Modified Eagle Medium (DMEM) supplemented with 10% fetal bovine serum (FBS) and 1% antibiotics at 37°C in a 5% CO_2_ atmosphere. After incubation for two days, differentiation medium (high-glucose DMEM, 10% FBS, 0.5 mΜ methylisobutylxanthine, 1 μΜ dexamethasone, and 5 μg/mL insulin) was added to the cells, followed by incubation for three days. Subsequently, the medium was replaced with high-glucose DMEM containing 10% FBS and 5 μg/mL insulin. The medium was changed every two days until the preadipocytes differentiated into white adipocytes, which were used in subsequent experiments.

### Establishment of insulin resistance model

The differentiated adipocytes were incubated in serum-free, high-glucose DMEM containing 10 ng/mL TNF-α and 0.2% bovine serum albumin (BSA) to induce insulin resistance [14].

### 3-(4,5-Dimethylthiazol-2-yl)-2,5-diphenyltetrazolium bromide (MTT) assay

Cell viability was determined by performing an MTT assay. Briefly, the cells were seeded in 96-well plates at 2 × 10^4^ cells/well, followed by incubation for 24 h. Next, 20 μL of MTT solution (5 mg/mL) was added to the cells, after which the mixture was incubated for another 4 h. The supernatant was replaced with dimethyl sulfoxide to lyse the cells. The absorbance of MTT was measured at 570 nm [15].

### Oil red O staining assay

Adipogenesis was determined in an oil red O staining assay as previously described [16]. The cells were washed with phosphate-buffered saline (PBS) three times and fixed in 10% formaldehyde for 30 min, after which they were washed again and dyed with oil red O solution for 30 min. After removing the remaining dye with 60% isopropanol, images were captured under an inverted microscope (Olympus, Tokyo, Japan).

### Enzyme-linked immunosorbent assay (ELISA)

IL-6, TNF-α, and IL-1β levels were measured by performing ELISA [17] (Elabscience, Wuhan, China). 3T3-L1 preadipocytes were centrifugated at 300 × *g* for 5 min, after which the supernatant was collected, loaded into 96-well plates, and incubated at 37°C for 1 h. Similar treatments were performed on five gradient concentrations of the standard. Subsequently, the medium was removed and horseradish peroxidase−conjugated secondary antibodies were added to the contents of the plates. The plates were incubated at 37°C for 30 min, after which 3,3′,5,5′-tetramethylbenzidine solution was added to their contents for 15 min. Lastly, the stop solution was added to terminate the reaction. A microplate reader (BioTek, Winooski, VT, USA) was used to measure absorbance at 450 nm, after which concentrations were calculated based on standard curves.

### Reverse transcription-polymerase chain reaction (RT-PCR) assay

TRIzol reagent was used to extract total RNA from adipocytes. RNA concentration was determined by measuring the optical density at 260 nm, followed by transcription into cDNA using PrimeScript RT Master Mix Kit (Takara Bio, Tokyo, Japan). SYBR Premix Ex Taq Kit (Takara Bio) was used to perform RT-PCR. The relative expression of target genes was calculated using the 2^−ΔΔCT^ method after normalization to glyceraldehyde 3-phosphate dehydrogenase (GAPDH) expression [18].

### Luciferase activity of the nuclear factor kappa B (NF-κB) promoter

Cells were transfected with pNF-κB Luc reporter plasmid (Beyotime, Shanghai, China) and Lipofectamine 3000 (Thermo Fisher Scientific, Waltham, MA, USA), followed by incubation for 48 h. Reporter activity was determined using a luciferase reporter assay kit (BioVision Inc., Los Angeles, CA, USA) by measuring fluorescence intensity using a synergy microplate reader (Bio-Rad, Hercules, CA, USA) [19].

### Immunofluorescence assay

Adipocytes were washed with PBS three times and fixed in 4% paraformaldehyde for 15 min. The cells were then mixed with 0.5% Triton X-100 in PBS for 20 min at room temperature and incubated with 5% BSA to be blocked for 30 min. The primary antibody against NF-κB p65 (1:200, ab201340, Abcam, Cambridge, UK) was added to the cells, after which the mixture was incubated at 37°C for 3 h. Subsequently, the cells were incubated with secondary antibody (1:200; Lot01321; CWBio, Beijing, China) at 37°C for 45 min. After three washes with PBS, 4′,6-diamidino-2-phenylindole was added to the samples, followed by incubation in the dark for 5 min and blocking with 50% glycerin. Lastly, images were captured under a fluorescence microscope (CKX53, OLYMPUS, Tokyo, Japan) [20].

## Western blotting

Western blotting was performed as previously described [21]. Following the extraction of total proteins from adipocytes using lysis buffer, a bicinchoninic acid kit (Cwbiotech, Beijing, China) was used to quantify the isolated proteins. Approximately 40 μg of protein was loaded and separated by 12% sodium dodecyl sulfate–polyacrylamide gel electrophoresis and then transferred onto polyvinylidene difluoride membrane (Millipore, Billerica, MA, USA). The membrane was incubated with 5% BSA (Solarbio, Beijing, China) followed by incubation with primary antibodies against inhibitor of nuclear factor kappa-B kinase (IKK) subunit beta, p-IKK, p-p65, p65, peroxisome proliferator-activated receptor gamma (PPAR-γ), CCAAT enhancer-binding protein alpha (C/EBPα), sterol regulatory element-binding protein (SREBP)-1 c, cleaved caspase-3, insulin receptor substrate 1 (IRS-1), glucose transporter type 4 (GLUT4), and GAPDH (1:1000 in each instance; Abcam, Cambridge, UK). Subsequently, the membrane was incubated with a secondary antibody (1:2000, Abcam) at room temperature for 1.5 h. Lastly, the bands were visualized with electrochemiluminescence solution and the relative expression level of each target protein was quantified using ImageJ software.

### Statistical analysis

Data were analyzed using the GraphPad Prism software (GraphPad Software Inc., San Diego, CA, USA) and have been presented as mean ± standard deviation. One-way analysis of variance was used to compare data among groups, whereas *t*-test was used to compare two independent data. P values <0.05 were considered statistically significant.

## Results

We hypothesized that BBR and Rb1 would have a synergetic inhibitory effect on TNF-α-induced inflammation in adipocytes. We first achieved the optimized incubation concentrations of BBR and Rb1 in adipocytes using the MTT assay. Afterward, we investigated the effects of BBR combined with Rb1 on cell proliferation, inflammation state, adipogenesis, and insulin resistance in TNF-α-treated adipocytes. Additionally, we evaluated the inhibitory effects of BBR combined with Rb1 on the NF-κB pathway in TNF-α-treated adipocytes. Lastly, the regulatory mechanisms underlying the effects of BBR+Rb1 on cell proliferation, inflammation, adipogenesis, and insulin resistance in the TNF-α-treated adipocytes were studied by introducing RANKL, which is an agonist of the NF-κB pathway.

### Determination of the optimum concentrations of BBR and Rb1

**Figure 1.**
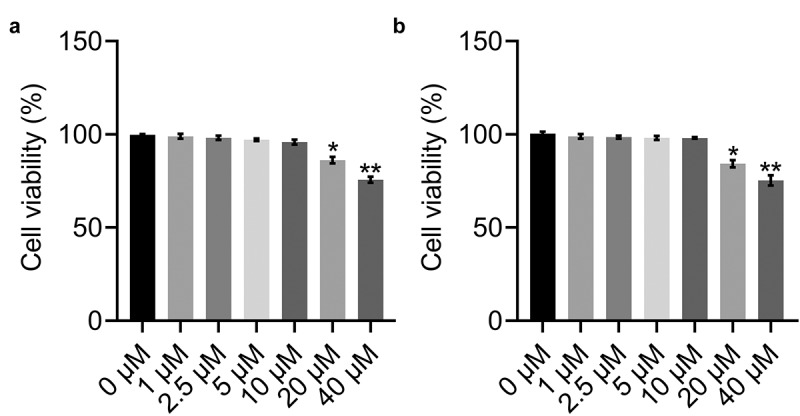
Viability of adipocytes treated with different concentrations of (a) BBR and (b) Rb1. Cell viability was determined by performing MTT assays. * indicates p < 0.05 and ** indicates p < 0.01 when data are compared to those obtained at 0 μM

To obtain the optimized concentrations of BBR and Rb1 in adipocytes, the cells were incubated with different concentrations of BBR (0, 1, 2.5, 5, 10, 20, and 40 μM) and Rb1 (0, 1, 2.5, 5, 10, 20, and 40 μM), followed by the assessment of cell viability using the MTT assay. As shown in Figure 1(a), BBR did not significantly affect cell viability over a concentration range of 0–10 μM. However, cell viability declined significantly when the concentration exceeded 20 μM. Similar results were obtained for Rb1 (Figure 1(b)). Therefore, BBR and Rb1 were used at a concentration of 10 μM each in the subsequent in vitro experiments.

### Proliferation and inflammation of TNF-α-treated adipocytes were synergistically decreased by BBR and Rb1

**Figure 2.**
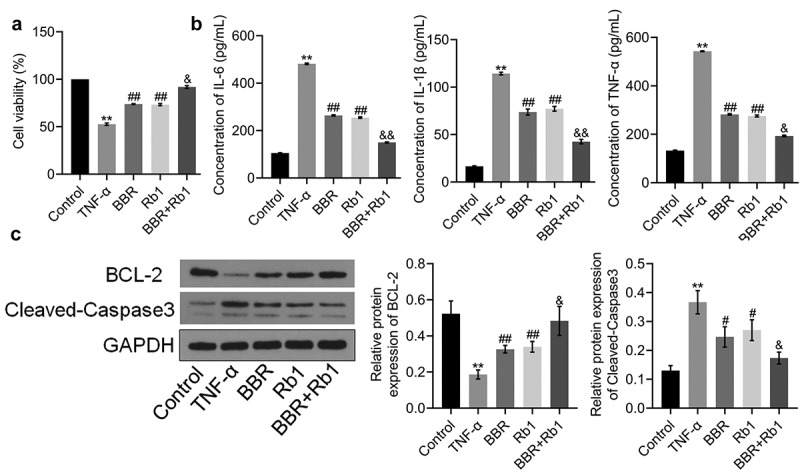
Synergistic effects of BBR and Rb1 on inflammation and the proliferation of TNF-α-treated adipocytes. (a) Cell viability was determined via MTT assay (**, p < 0.01, compared to Control; ##, p < 0.01, compared to TNF-α; &, p < 0.05, compared to BBR or Rb1). (b) IL-6, IL-1β, and TNF-α levels were measured by performing ELISA (**, p < 0.01, compared to Control; ##, p < 0.01, compared to TNF-α; &, p < 0.05, compared to BBR or Rb1; &&, p < 0.01, compared to BBR or Rb1). (c) The expression levels of Bcl-2 and cleaved caspase-3 were determined in western blot assays (**, p < 0.01, compared to Control; #, p < 0.05, compared to TNF-α; ##, p < 0.01, compared to TNF-α; &, p < 0.05, compared to BBR or Rb1; &&, p < 0.01, compared to BBR or Rb1)

To explore the synergistic protective effect of BBR and Rb1 on TNF-α-treated adipocytes, differentiated adipocytes were incubated with blank medium (Control group), 10 ng/mL TNF-α (TNF-α group), 10 ng/mL TNF-α and 10 μM BBR (BBR group), 10 ng/mL TNF-α and 10 μM Rb1 (Rb1 group), or 10 ng/mL TNF-α, 5 μM BBR, and 5 μM Rb1 (BBR+Rb1 group). As shown in Figure 2(a), the viability of cells in the TNF-α group was significantly low compared to that of cells in the Control group. However, cell viability was considerably increased by BBR, Rb1, and BBR+Rb1. Additionally, the viability of cells in the BBR+Rb1 group was higher than that in the BBR group or Rb1 group.

ELISA was performed to assess the production of inflammatory factors. As shown in Figure 2(b), IL-6 concentration significantly increased from 105.29 pg/mL in the Control group to 481.78 pg/mL in the TNF-α group; however, it was considerably decreased afterward to 264.05 pg/mL and 254.99 pg/mL by BBR and Rb1, respectively, and to 150.06 pg/mL by BBR and Rb1 together. IL-1β concentration was found to be 16.52, 114.29, 73.78, 77.20, and 42.59 pg/mL in the Control, TNF-α, BBR, Rb1, and BBR+Rb1 groups, respectively. In addition, TNF-α concentration was increased from 132.83 pg/mL in the Control group to 543.03 pg/mL in the TNF-α group but was significantly decreased afterward to 282.50 pg/mL and 275.65 pg/mL by BBR and Rb1, respectively, and to 193.09 pg/mL by BBR+Rb1.

In the next experiment, we evaluated the expression levels of proteins involved in apoptosis in the treated adipocytes. As shown in Figure 2(c), TNF-α significantly downregulated the expression of B-cell lymphoma 2 (Bcl-2) but significantly upregulated that of cleaved caspase-3. These changes were considerably reversed by BBR, Rb1, and BBR+Rb1; however, the effects of BBR+Rb1 were higher than those of BBR or Rb1 alone.

### Adipogenesis and insulin resistance in TNF-α-treated adipocytes were synergistically suppressed by BBR and Rb1

**Figure 3.**
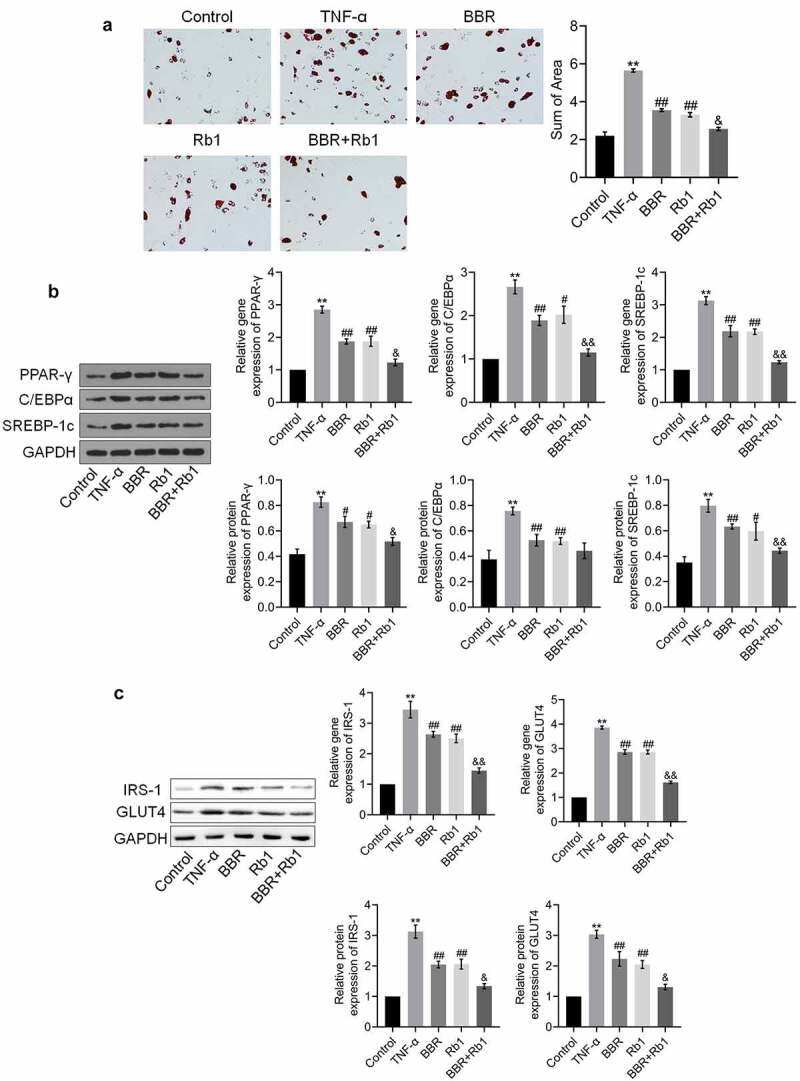
BBR and Rb1 synergistically suppressed adipogenesis and insulin resistance in TNF-α-treated adipocytes. (a) Adipogenesis was evaluated via oil red O staining assay (**, p < 0.01, compared to Control; ##, p < 0.01, compared to TNF-α; &, p < 0.05, compared to BBR or Rb1). (b) The gene and protein expression levels of PPAR-γ, C/EBPα, and SREBP-1 c were measured by performing RT-PCR and western blot assays, respectively (**, p < 0.01, compared to Control; ##, p < 0.01, compared to TNF-α; &&, p < 0.01, compared to BBR or Rb1). (c) The gene and protein expression levels of IRS-1 and GLUT4 were measured via RT-PCR and western blot assays, respectively (**, p < 0.01, compared to Control; ##, p < 0.01, compared to TNF-α; &, p < 0.05, compared to BBR or Rb1; &&, p < 0.01, compared to BBR or Rb1)

The results of the oil red O staining assay shown in Figure 3(a) indicate that the sum of area was significantly increased after the cells were stimulated with TNF-α but was substantially decreased in the BBR and Rb1 groups. Additionally, the sum of area was lower in the BBR+Rb1 group than in the BBR group or Rb1 group. In the next experiment, we assessed the expression levels of adipogenesis-related genes and proteins. As shown in Figure 3(b), the expression levels of PPAR-γ, C/EBPα, and SREBP-1 c were significantly higher in the TNF-α group than in the Control group but were drastically decreased by BBR and Rb1. Furthermore, the expression levels of PPAR-γ, C/EBPα, and SREBP-1 c were lower in the BBR+Rb1 group than in the BBR group or Rb1 group.

The expression levels of IRS-1 and GLUT4 are indicative of the state of insulin resistance. As shown in Figure 3(c), the expression levels of IRS-1 and GLUT4 were considerably increased by TNF-α but greatly decreased by BBR, Rb1, and BBR+Rb1; however, the effects of BBR+Rb1 were higher than those of BBR or RB1.

### BBR and Rb1 synergistically inhibit the NF-κB pathway in TNF-α-treated adipocytes

**Figure 4.**
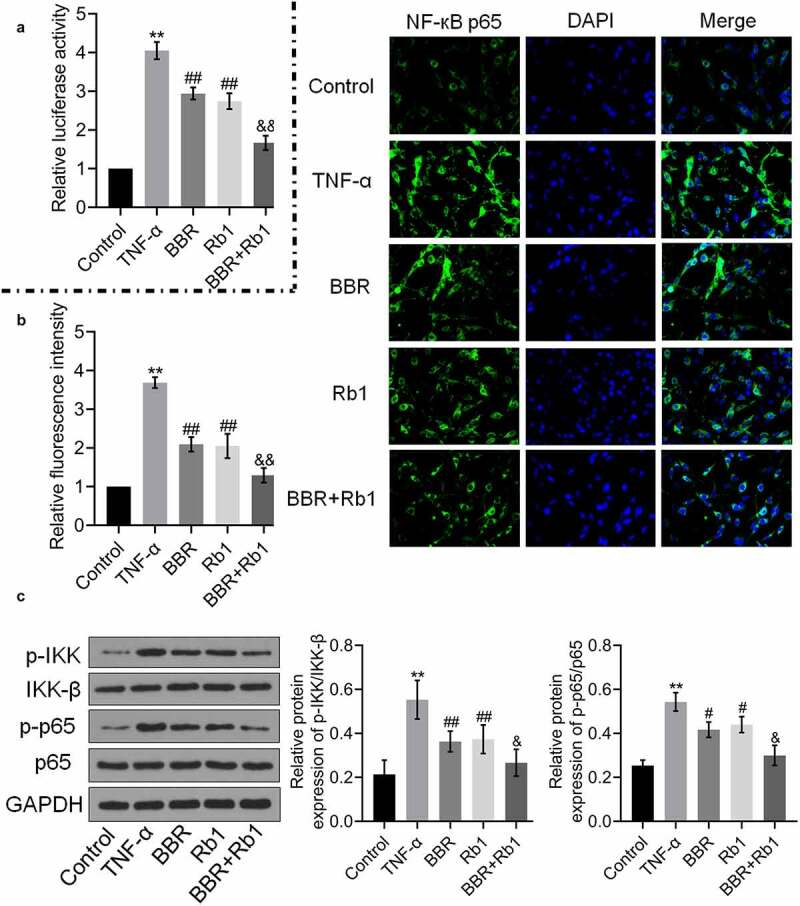
BBR and Rb1 synergistically inhibited the NF-κB pathway in TNF-α-treated adipocytes. (a) The transcriptional activity of NF-κB was evaluated by determining the luciferase activity of NF-κB promoter (**, p < 0.01, compared to Control; ## p < 0.01, compared to TNF-α; &&, p < 0.01, BBR or Rb1). (b) The expression level of NF-κB p65 was evaluated in an immunofluorescence assay (**, p < 0.01, compared to Control; ##, p < 0.01, compared to TNF-α; &&, p < 0.01, compared to BBR or Rb1). (c) The expression levels of p-IKK, IKK-β, p-p65, and p65 were measured in western blot assays (**, p < 0.01, compared to Control; #, p < 0.05, compared to TNF-α; ##, p < 0.01, compared to TNF-α; &, p < 0.05, compared to BBR or Rb1)

To explore the mechanism underlying the anti-inflammatory effect of BBR+Rb1, NF-κB activity was evaluated in each group. As shown in Figure 4(a-b), relative luciferase activity and the relative fluorescence intensity of NF-κB p65 were significantly increased by TNF-α but greatly decreased by BBR, Rb1, and BBR+Rb1. However, the effects of BBR+Rb1 were considerably higher than those of BBR or Rb1.

Additionally, as shown in Figure 4(c), the relative expression levels of p-IKK/IKK-β and p-p65/p65 were markedly higher in the TNF-α group than in the control group but were reduced by BBR, Rb1, and BBR+Rb1, with the effect of BBR+Rb1 being higher than that of BBR or Rb1.

### RANKL inhibited the effects of BBR+Rb1 on the proliferation and inflammation of TNF-α-treated adipocytes

**Figure 5.**
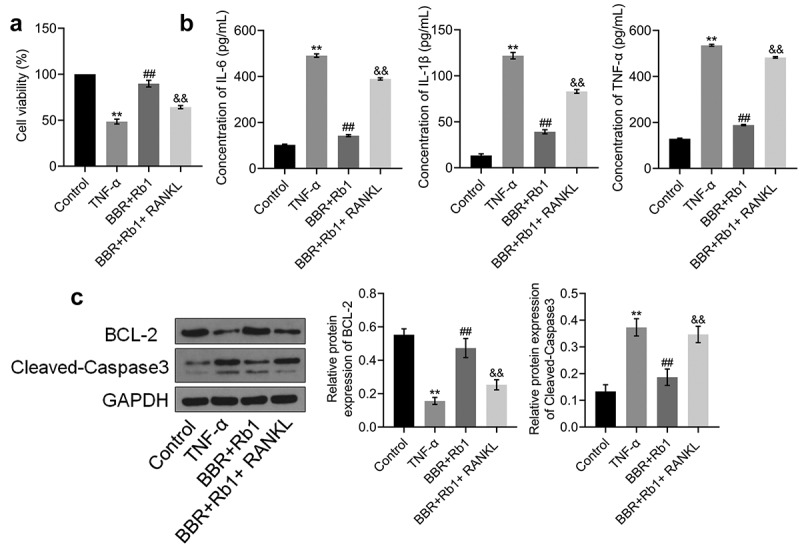
RANKL inhibited the effects of BBR+Rb1 on inflammation and the proliferation of TNF-α-treated adipocytes. (a) Cell viability was determined via an MTT assay (**, p < 0.01, compared to Control; ##, p < 0.01, compared to TNF-α; &&, p < 0.01, compared to BBR+Rb1). (b) IL-6, IL-1β, and TNF-α levels in the different groups were measured be performing ELISA (**, p < 0.01, compared to Control; ##, p < 0.01, compared to TNF-α; &&, p < 0.01, compared to BBR+Rb1). (c) The expression levels of Bcl-2 and cleaved caspase-3 were determined in western blot assays (**, p < 0.01, compared to Control; ##, p < 0.01, compared to TNF-α; &&, p < 0.01, compared to BBR+Rb1)

We investigated whether the anti-inflammatory effect of BBR+Rb1 was mediated via the NF-κB pathway by co-incubating the cells with RANKL (a natural agonist of the NF-κB pathway) and BBR+Rb1. In the experiment, adipocytes were incubated with blank medium (Control group); 10 ng/mL TNF-α (TNF-α group); 10 ng/mL TNF-α, 5 μM BBR, and 5 μM Rb1 (BBR+Rb1 group); or 10 ng/mL TNF-α, 5 μM BBR, 5 μM Rb1, and 100 ng/mL RANKL [22] (BBR+Rb1+ RANKL group). As shown in Figure 5(a), cell viability was significantly lower in the TNF-α group than in the Control group but was greatly increased after co-incubation with BBR and Rb1. Moreover, cell viability was decreased more by RANKL than by BBR+Rb1.

As shown in Figure 5(b), IL-6 concentration was significantly increased from 102.90 pg/mL to 490.66 pg/mL by TNF-α but was substantially decreased to 142.98 pg/mL after the cells were co-treated with BBR and Rb1. Furthermore, compared to BBR+Rb1, RANKL markedly reversed the effect of TNF-α on IL-6 secretion. Specifically, RANKL increased IL-6 concentration to 389.11 pg/mL. IL-1β concentration was found to be 13.39, 121.90, 39.17, and 83.07 pg/mL in the Control, TNF-α, BBR+Rb1, and BBR+Rb1+ RANKL groups, respectively. In addition, TNF-α concentration was considerably increased from 129.31 pg/mL to 535.42 pg/mL after the cells were stimulated with TNF-α but was significantly suppressed to 188.59 pg/mL by BBR+Rb1. TNF-α concentration was drastically increased to 482.91 pg/mL in the BBR+Rb1+ RANKL group.

The expression levels of apoptosis-related proteins in the treated adipocytes were also evaluated in the study. As shown in Figure 5(c), Bcl-2 expression was significantly downregulated, whereas the expression of cleaved caspase-3 was significantly upregulated in the TNF-α group. However, these effects were reversed after the co-treatment with BBR and Rb1. The results showed that the addition of RANKL resulted in a higher suppression of Bcl-2 expression and a higher increase in the expression of cleaved caspase-3 compared to the effects of only BBR+Rb1.

### RANKL inhibited the effects of BBR+Rb1 on adipogenesis and insulin resistance in TNF-α-treated adipocytes

**Figure 6.**
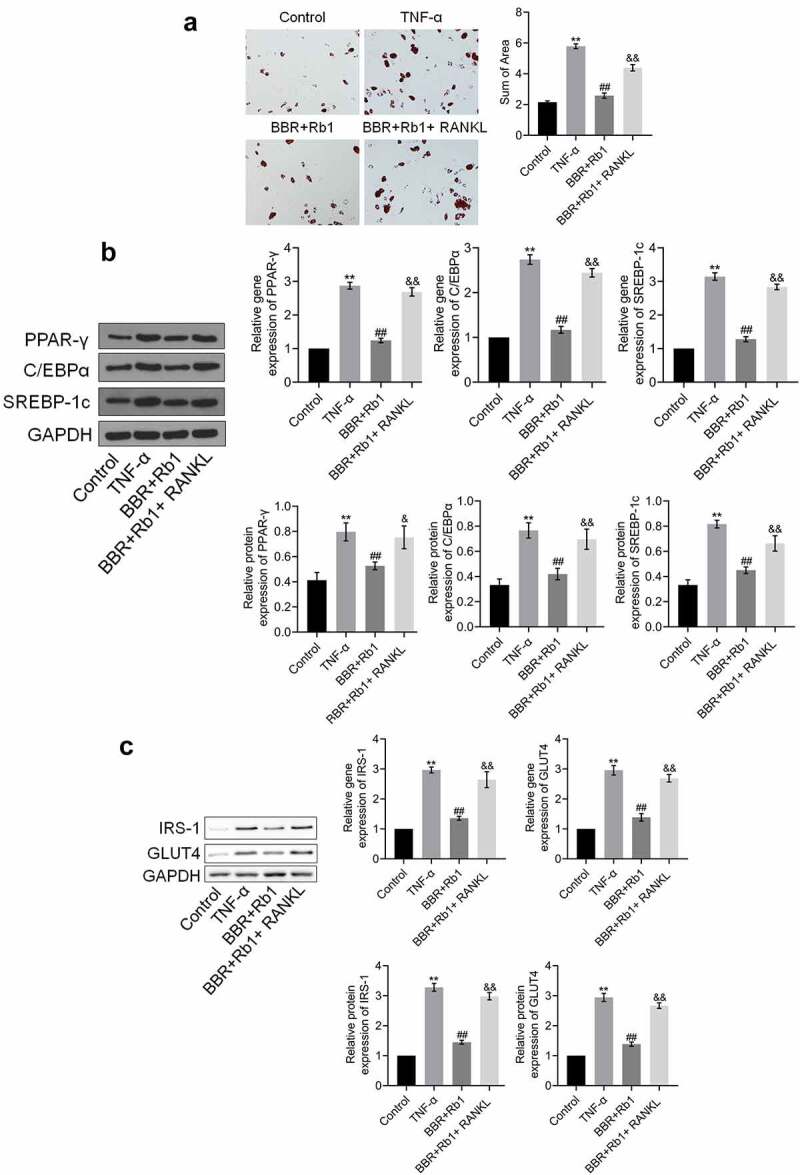
RANKL inhibited the effects of BBR+Rb1 on adipogenesis and insulin resistance in TNF-α-treated adipocytes. (a) Adipogenesis was evaluated by performing oil red O staining assay (**, p < 0.01, compared to Control; ##, p < 0.01, compared to TNF-α; &&, p < 0.05, compared to BBR+Rb1). (b) The gene and protein expression levels of PPAR-γ, C/EBPα, and SREBP-1 c were measured in RT-PCR and western blot assays, respectively (**, p < 0.01, compared to Control; ##, p < 0.01, compared to TNF-α; &&, p < 0.05, compared to BBR+Rb1). (c) The gene and protein expression levels of IRS-1 and GLUT4 were measured in RT-PCR and western blot assays, respectively (**, p < 0.01, compared to Control; ##, p < 0.01, compared to TNF-α; &&, p < 0.01, compared to BBR+Rb1)

We evaluated the state of adipogenesis after the cells were treated with RANKL. As shown in Figure 6(a), the sum of area significantly increased after the treatment with TNF-α; however, it was considerably decreased in the BBR+Rb1 group. Moreover, the sum of area was higher in the BBR+Rb1+ RANKL group than in the BBR+Rb1 group.

The results also showed that the expression levels of PPAR-γ, C/EBPα, and SREBP-1 c were significantly increased by TNF-α but were decreased after the co-treatment with BBR and Rb1. Additionally, the expression levels of PPAR-γ, C/EBPα, and SREBP-1 c were markedly increased by RANKL (Figure 6(b)).

We further evaluated the expression of proteins involved in insulin resistance to assess the effects of the various agents. As shown in Figure 6(c), the expression levels of IRS-1 and GLUT4 were markedly higher in the TNF-α group than in the Control group; however, they were considerably decreased after the co-treatment with BBR and Rb1. Additionally, compared to the BBR+Rb1 group, the group treated with RANKL showed a markedly higher upregulation of IRS-1 and GLUT4 expression.

### RANKL inhibited the effects of BBR+Rb1 on the NF-κB pathway in TNF-α-treated adipocytes

**Figure 7.**
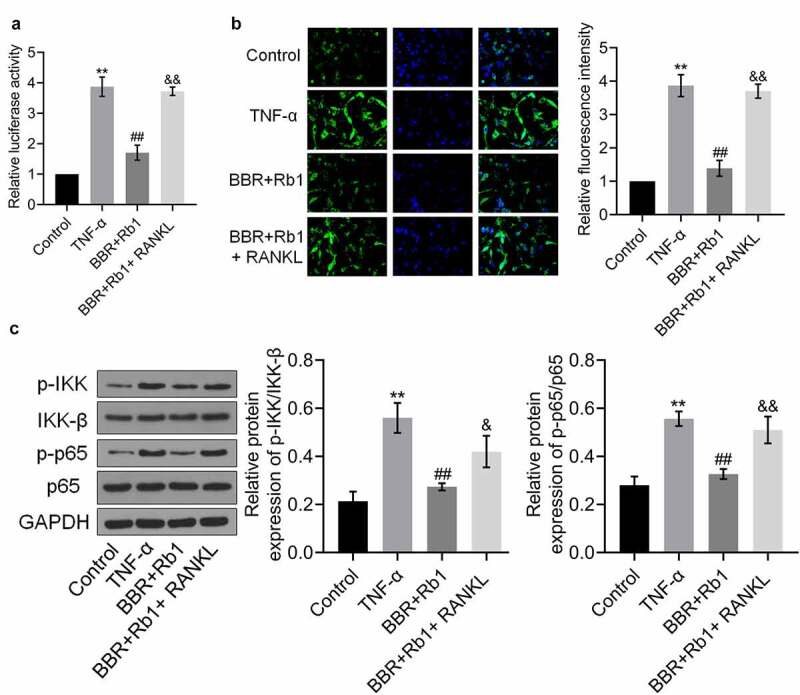
RANKL inhibited the effects of BBR+Rb1 on the NF-κB pathway in TNF-α-treated adipocytes. (a) The transcriptional activity of NF-κB was evaluated by measuring the luciferase activity of NF-κB promoter (**, p < 0.01, compared to Control; ##, p < 0.01, compared to TNF-α; &&, p < 0.01, compared to BBR+Rb1). (b) The expression level of NF-κB p65 was evaluated in an immunofluorescence assay (**, p < 0.01, compared to Control; ##, p < 0.01, compared to TNF-α; &&, p < 0.01, compared to BBR+Rb1). (c) The expression levels of p-IKK, IKK-β, p-p65, and p65 were determined in western blot assays (**, p < 0.01, compared to Control; ##, p < 0.01, compared to TNF-α; &&, p < 0.01, compared to BBR+Rb1)

As shown in Figure 7(a-b), the relative luciferase activity and fluorescence intensity of NF-κB p65 were significantly increased in the TNF-α group but markedly decreased in the BBR+Rb1 group. Furthermore, relative luciferase activity and the relative fluorescence intensity of NF-κB p65 were markedly higher in the BBR+Rb1+ RANKL group than in the BBR+Rb1 group.

Finally, Figure 7(c) shows that the relative expression levels of p-IKK/IKK-β and p-p65/p65 were markedly higher in the TNF-α group than in the Control group, but were decreased after the co-treatment with BBR and Rb1. Additionally, the expression levels of p-IKK/IKK-β and p-p65/p65 were higher in the BBR+Rb1+ RANKL group than in the BBR+Rb1 group.

## Discussion

Obesity is defined as the excessive accumulation of fat transformed from superfluous energy. It has been reported by the World Health Organization that a BMI that is higher than 30 kg/m^2^ indicates obesity [23]. The quickening pace of life and changes in diet cause increased morbidity annually as a result of obesity [24]. It was recently reported that chronic inflammation is involved in the pathogenesis of obesity [25]. The hypothalamic-pituitary-adrenal axis and the autonomic nervous system are usually activated to prevent the development of obesity. However, this induces an increase in glucocorticoid production to trigger the differentiation of preadipocytes. Consequently, the growth of white adipose tissue is accelerated. During this vicious circle, there is an excessive release of proinflammatory factors such as TNF-α and IL-6, which finally results in the induction of chronic inflammation in adipose tissues [26].

In the present study, an insulin resistance model was established in adipocytes by incubating the cells with TNF-α. This model has been widely used to investigate adipogenesis and lipolysis in different studies [14,27]. Our results showed that TNF-α decreased the viability of the adipocytes. Additionally, it increased adipogenesis as well as the production of inflammatory factors. The effects of TNF-α are consistent with those obtained in previous studies [28]. We also found that BBR and Rb1, as individual agents, significantly ameliorated proliferation, inflammation, and adipogenesis in the TNF-α-treated adipocytes, which are consistent with previously reported effects of BBR [29]. Interestingly, a synergetic effect was observed after treating the cells with 5 μM BBR and 5 μM Rb1 compared to 10 μM BBR or 10 μM Rb1. Therefore, we believe that BBR and Rb1 can be combined in future clinical therapeutic strategies for obesity management to achieve improved anti-obesity effects of the two compounds.

NF-κB is a transcriptional factor that is widely distributed in different types of cells. It plays a role in inflammatory reactions by regulating the expression levels of cytokines, chemokines, and adhesion molecules [30]. Under normal physiological conditions, NF-κB is located in the cytoplasm, where it remains inactive by binding to its inhibitor IκB. The kinase of IκB (IKK) can be activated by inflammatory factors and during ischemic stress. IKK is a complex consisting of three subunits: IKK-α, IKK-β, and IKK-γ. It is reported that IKK-β is the essential subunit for the activation of the NF-κB signaling pathway. Consequently, it is an important mediator for multiple proinflammatory elements to activate NF-κB. Once IKK-β is activated, IκB phosphorylation is induced; this leads to the inactivation of IκB, which further disassociates from NF-κB to induce the transfer of NF-κB from the cytoplasm into the nucleus. Consequently, the transcription of different inflammatory factors is activated by NF-κB p65, a variant of NF-κB in the nucleus, which finally triggers the development of chronic inflammation in adipose tissues [31].

In the present study, we found that the NF-κB signaling pathway was significantly activated in TNF-α-treated adipocytes, which was consistent with previous findings [32]. To investigate whether the anti-inflammatory effect of BBR+Rb1 was mediated via inhibition of the NF-κB signaling pathway, TNF-α-stimulated adipocytes were treated with RANKL, which is a natural activator of NF-κB [33], and BBR+Rb1. Interestingly, the effects of BBR+Rb1 on inflammation, adipogenesis, and the proliferation of the TNF-α-treated adipocytes were significantly inhibited by RANKL. This indicated that the therapeutic effects of BBR+Rb1 on TNF-α-treated adipocytes were mediated via suppression of the NF-κB signaling pathway. In our future work, the regulatory effects of BBR+Rb1 on the upstream pathway of NF-κB signaling will be further investigated to better understand the mechanism underlying the potential anti-obesity effect of BBR+Rb1.

## Conclusion

Our study revealed that BBR and Rb1 have a synergetic protective effect against TNF-α-induced inflammation in adipocytes through inhibition of the NF-κB signaling pathway.

## Research highlights

BBR and Rb1 have a synergetic effect on adipogenesis.

BBR and Rb1 have a synergetic effect on inflammation in adipocytes.

BBR and Rb1 have a synergetic inhibitory effect on the NF-κB signaling pathway.
